# Editorial: Obesity and sustainability

**DOI:** 10.3389/fnut.2023.1235036

**Published:** 2023-06-22

**Authors:** Melissa Pflugh Prescott, Tarek Ben Hassen, Hamid El Bilali

**Affiliations:** ^1^Department of Nutrition, School of Medicine, Case Western Reserve University, Cleveland, OH, United States; ^2^Program of Policy, Planning, and Development, Department of International Affairs, College of Arts and Sciences, Qatar University, Doha, Qatar; ^3^International Centre for Advanced Mediterranean Agronomic Studies (CIHEAM-Bari), Bari, Italy

**Keywords:** nutrition security, obesity, sustainable development, public health nutrition, sustainable agriculture, food insecurity

The prevalence of overweight and obesity is high globally, both among adults and children. Moreover, the prevalence of obesity exceeds that of underweight, which implies an increased risk of the non-communicable diseases (NCDs) associated with elevated body mass index (BMI), e.g., cardiovascular diseases and type 2 diabetes. Therefore, obesity continues to be one of the most pressing global health challenges. However, evidence shows that obesity is more than just a health and nutritional issue; it is a sustainable development issue with social, environmental, ethical, and economic determinants and implications. Despite that, the existing scholarly literature centers obesity as a dietary and health issue. The growing recognition of the association between food insecurity and obesity has prompted a shift toward prioritizing nutrition security, which occurs when all individuals have access to sufficient and consistent food that supports optimal health and wellbeing in a manner that does not undermine one's cultural foodways. Accordingly, this Research Topic presents a compilation of papers that collectively examine the complex relationship between obesity and sustainability. These eight articles explicate the obesity-sustainability nexus: socio-economic factors and determinants, the role of culture in food waste behaviors, and the use of nutrition-sensitive diets and agriculture in addressing these public health priorities.

Regarding obesity disparities, Munir et al. investigated the relationship between global obesity and the global social index. They concluded that obesity is most prevalent in medium human development than in low and very high development levels. This may be because this group is most prone to the impacts of increased availability of ultra-processed foods and their aggressive marketing, as they have increased buying capacity for these foods and subsequently more disruptions to their traditional foodways. In addition, very high human development societies tend to prioritize their health, such as eating a more balanced diet and being more physically active.

In Bangladesh, Mazumder et al. examined the nutritional status of young adults in saline-prone coastal areas. They concluded that employment was associated with being overweight. This finding aligns with those of Munir et al., as employed Bangladeshi adults may have a greater ability to buy ultra-processed meals, placing them at higher risk of weight gain. Moreover, because Bangladesh's agrobiodiversity has declined due to increasing soil salinity caused by rising sea levels, traditional foodways have been disturbed, which may further increase the likelihood of processed food consumption. Mazumder et al. also concluded that female, but not male, employment was associated with reduced underweight. This finding is related to Li et al.'s regarding the obesity prevalence among Chinese youth. Indeed, Li et al. found that maternal education, and not paternal, is associated with reduced obesity among children aged 7 to 18. These findings suggest the potential role of addressing disparities in female education and workforce development on health equity.

Further, differences in dietary quality are often at the core of socio-economic, racial, and ethnic health disparities. People lacking food sufficiency are generally ill-equipped to reach their full health potential as they lack access to the foods required to prevent or manage chronic disease. One group at high risk for food insufficiency is the elderly. Huang et al. explored the frequency of Beijing older adults consuming three meals per day. They concluded that most older adults consume all three meals, mostly at home. However, the differences in meal frequency by monthly household income, alcohol drinking status, and daily physical activity suggest some population sub-groups should be targeted for food assistance to promote a healthy aging society. However, sustainable development requires nutrition security, not just food sufficiency. The findings of Yang et al. can be extrapolated to suggest what types of food should be prioritized to reduce obesity via nutrition security. They conclude that diets inclusive of a complex of 11 antioxidant micronutrients were inversely associated with obesity prevalence, and diets high in iron and vitamin C may make the most significant contribution to reduced obesity. Fu et al. further elucidated the role of specific dietary compounds in addressing obesity by investigating a potential mechanism to explain the anti-obesity properties of resistant starch using an animal model. They supplemented the diet of high-fat-fed Sprague-Dawley rats with unripe plantain flour. They concluded that unripe plantain powder positively impacted gut microbiota, altered glucolipid metabolism gene expression, and decreased insulin levels. Taken together, the latter two studies suggest that the future of population health guidance to treat obesity may extend beyond the general promotion of healthy diets and evolve into a “food as medicine approach,” where foods high in specific micronutrients and bioactive are used to ameliorate this disease.

Finally, improving agricultural yields and reducing food waste are two key strategies to reduce greenhouse gas emissions. However, it is important that initiatives facilitating a more efficient food system do not exacerbate obesity risks. Guardiola-Márquez and Jacobo-Velázquez conducted a review to understand better the potential of biofertilization and nano-fertilization practices to improve the concentrations of bioactive compounds in anti-obesogenic plants. These practices may increase the concentrations of flavonoids, phenolic acids, polyphenols, and other bioactive compounds that may, in turn, improve public health through the modulation of gut microbiota and suppression of obesity-induced inflammation while also improving plant growth and resilience. Hassan et al. explored the relationship between religiosity and food waste. They concluded that religiosity was inversely related to food waste. This suggests that public campaigns to reduce food waste and overconsumption should be culturally tailored, as culture is related to people's food values and perceptions.

Collectively, these eight articles provide a comprehensive overview of the multifaceted and multidimensional relationship between obesity and sustainability. They emphasize the need for integrated, holistic, and multidisciplinary approaches considering the interconnectedness of human health, the environment, and societal wellbeing. This collection of papers elucidates the role of nutrition-focused solutions in sustainable development may stimulate further research, policy, and community engagement and action on the obesity and sustainability nexus.

## Author contributions

MP drafted the initial manuscript. All authors revised it critically and reviewed the final version.

